# Comment on López-Yerena et al. “Absorption and Intestinal Metabolic Profile of Oleocanthal in Rats” *Pharmaceutics* 2020, *12*, 134

**DOI:** 10.3390/pharmaceutics12080720

**Published:** 2020-07-31

**Authors:** Amal Kaddoumi, Khalid El Sayed

**Affiliations:** 1 Department of Drug Discovery & Development, Harrison School of Pharmacy, Auburn University, 720 S. Donahue Drive, Auburn, AL 36849, USA; 2 School of Basic Pharmaceutical and Toxicological Sciences, College of Pharmacy, University of Louisiana at Monroe, 1800 Bienville Drive, Monroe, LA 71201, USA

**Keywords:** oleocanthal, metabolism, pharmacokinetics, intestinal absorption

## Abstract

This comment is intended to discuss errors observed in the title paper, doi:10.3390/pharmaceutics12020134. When this paper was published, the authors of this commentary were excited to read it. However, the more we read, the more pitfalls were observed, which necessitated a response to revise the many errors and misleading information included in this publication.

## 1. Introduction

*S*-(-)-Oleocanthal (OLC) is an exceptional natural phenolic compound exclusively occurring in extra-virgin olive oil (EVOO). It is attracting the attention of several research groups due to its documented activities against cancer, inflammation, and Alzheimer’s disease. Pharmacokinetics of this unique natural product has been very challenging to olive phenolic researchers due to the exceptional chemistry of the molecule. 

## 2. Discussion

The authors of López-Yerena et al. (2020) [1] neither declared the purity of the used (-)-oleocanthal by any analytical technique nor did they attempt to confirm its identity by any chromatographic and/or spectroscopic tools. This is very important due to the high instability and sensitivity of *S*-(-)-oleocanthal to air, light, and moisture, and the close structural similarity of several related secoiridoids present in EVOO. Commercially available oleocanthal may contain impurities of other related olive phenolics, such as hydroxyoleocanthal, which could be assumed as one of oleocanthal metabolites if not thoroughly evaluated. Thus, the purity of oleocanthal should have been checked and reported. In addition, during the purification process, there is a high possibility of artifact transformation of the *E*-geometry in the *S*-(-)-oleocanthal to the *Z*-geometrical isomer over time by extended air/light exposure or chromatographic purification on polar stationary phases. Therefore, there is an absolute need to declare multiple chromatographic and spectroscopic authentication methods to ratify the *E-S*-(-)-oleocanthal identity and purity. Lack of reporting this confirmatory data would question the credibility of subsequent study results and conclusions. 

**Figure 1:**
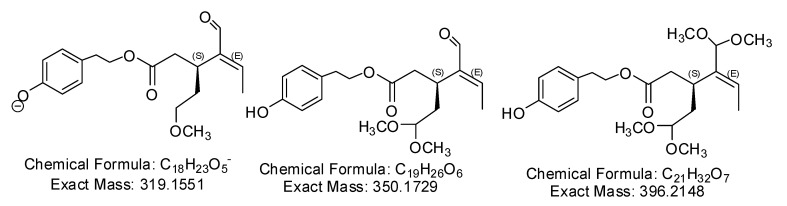
Acetalization products of (-)-oleocanthal C-1 and C-3 aldehydes by reaction with methanol.

**Figure 2:**
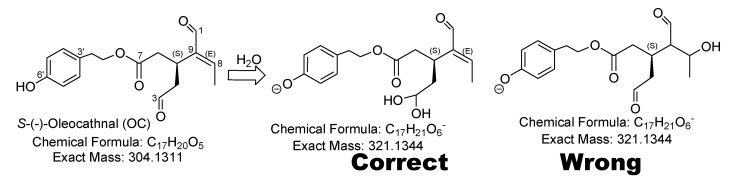
Revision of the metabolite B structure.

The use of methanol in the extraction and purification of (-)-oleocanthal and its metabolites is inappropriate because it is well-documented in literature that (-)-oleocanthal reactive aldehyde C-3 will promptly form the hemiacetal and acetal [2,3,4], followed by the less reactive aldehyde C-1 tendency to form the same products, as shown in Figure 1. Hemiacetal and acetal formation could significantly minimize, if not nullifying the availability of (-)-oleocanthal, which will ultimately complicate the detection of (-)-oleocanthal and related metabolites. Additionally, the structure shown for metabolite B in the title paper [1] is an inaccurate interpretation for the correct gem-diol analog of (-)-oleocanthal, which is formed because of water addition to the C-3 aldehyde, as shown in Figure 2 and reports in the literature [2,3,4]. The only way to confirm the listed structure B is to run detailed spectroscopic analyses including 2D-NMR experiments, like HMBC or 2D-INADEQUATE and mass–mass fragmentation studies.

Next, one should question, “How come the more polar metabolites OLC+OH, OLC+H_2_O and their corresponding glucuronide metabolites to elute at longer retention times than or even close to (-)-oleocanthal on C18 reversed phase?”. The variance of different C18-reversed phase packing materials, particle sizes, elution rates, mobile systems, etc., may result in minor retention time variations when the chemical structures vary by one or two polar functional groups. However, this will not be true for a glucuronide conjugate with an additional three hydroxy groups and a carboxy functionality to overlap or elute at a close proximity to the parent (-)-oleocanthal retention time. These sort of errors immediately question the credibility of metabolite(s) identity interpretations.

**Figure 3:**
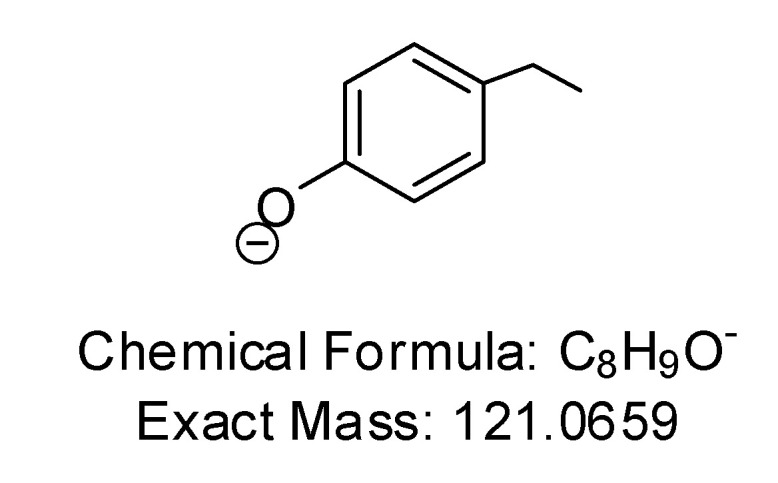
Diagnostic (-)-oleocanthal fragment at negative ion mode mass spectrometry.

It is not clear if the authors used specific software to filter out any non-oleocanthal metabolites and/or analog fragments. For example, the well-established fragment 4-hydroxy-ethylbenzene is a key diagnostic mass fragment for (-)-oleocanthal [4,5,6]. The authors did not explicitly indicate whether they used this fragment or any MS–MS fragmentation of any obtained peak to confirm the metabolites’ identity and correlate them with the tyrosol moiety of the parent (-)-oleocanthal via obtaining this diagnostic fragment, as shown in Figure 3. There was no interpretation of the fragments listed in Table 1 [1], nor any proposed potential fragmentation schemes, which clearly indicate mass–mass fragmentation was not used for metabolites identity confirmation. Furthermore, the authors did not explain why the metabolism will target breaking the α,β-unsaturated conjugation Δ^8,9^ system by hydration and hydrogenation and leave the highly reactive C-3 aldehyde unreacted [2,3,4]. While it is not clear if the authors detected any ester hydrolysis products, which is a very likely metabolic/chemical pathway expected to precede the Phase I and Phase II metabolic pathways. The exposed linear ester bond can be an easy target for esterase enzymatic or pH-induced hydrolysis.

Collectively, for metabolites identification, the authors should provide additional confirmatory evidence for their structures’ elucidation, including NMR, mass spectral, and/or chromatographic data.

Furthermore, the authors provided answers to each unexpected observation by speculation from unrelated reports in the literature, especially to justify observed metabolites. The conclusion of the study is not supported by the data provided. The article contained many errors; for example, the equations used to calculate effective permeability and corrected concentrations (Equations (1) and (2)) were not correct, which could provide an explanation of why LEV permeability was less than expected from reported values in the literature. This also raises suspicions about the values obtained for (-)-oleocanthal. The authors should have referred to Wahajuddin et al. (2012) for correct equations [7].

Moreover, the authors indicated that (-)-oleocanthal has “low solubility and relatively high lipophilicity”, which is again not correct. (-)-Oleocanthal and other olive phenolics can be better described as “amphiphilic” rather than lipophilic [8]. At the concentration used for this study, that is 0.1 mg/mL, (-)-oleocanthal is highly soluble in water and has no solubility problems [8], which should not be an issue or used to explain low absorption. (-)-Oleocanthal water solubility exceeds the range of 400–500 μg/mL [6,8]. Besides, the idea of the in-situ perfusion studies is to omit the solubility and evaluate the permeability. While the results provided do not support (-)-oleocanthal as a BCS class II compound, the authors’ discussion of (-)-oleocanthal BCS classification is confusing and not justified [1]. Did the authors conclude that (-)-oleocanthal is a BCS class II compound based on presented results, or based on its assumed physicochemical properties (low solubility and relatively high lipophilicity)? 

Under Section 2.3.3. Intestinal Perfusion, the authors stated: “*the outflow perfusate was collected in 1.5 mL amber vials at 5 min intervals for 60 min*”. With a flow rate of 1 mL/min, within 5 min it would be expected to collect 5 mL. Therefore, a 1.5 mL vial will not be suitable in this case. Besides, the used perfusion flow rate (1 mL/min) is fast and rarely used in such studies because it would reduce the mean residence time of compounds in the intestinal lumen, which could affect the degree of absorption and thus results interpretation. 

In their conclusion, the authors noted: “*However, previous research has indicated that higher levels of OLC reach human plasma than in rats [11]*”. Yet, the stated reference, Lennernäs 1997, did not evaluate (-)-oleocanthal permeability and absorption; thus, to jump to the conclusion that in humans OLC will be absorbed higher without justification is not valid [9].

## 3. Conclusions

The paper published by López-Yerena et al. 2020 in *Pharmaceutics* is marred by several serious scientific flaws and lacks scientific rigors, which question the credibility of this study results and conclusions.
